# Supplementary figures and images for: An enhancer screen identifies new suppressors of small-RNA-mediated epigenetic gene silencing
Source: PLoS Genet. 2021 Jun 22;17(6):e1009645. doi: 10.1371/journal.pgen.1009645 (PMC8253403; doi:10.1371/journal.pgen.1009645)

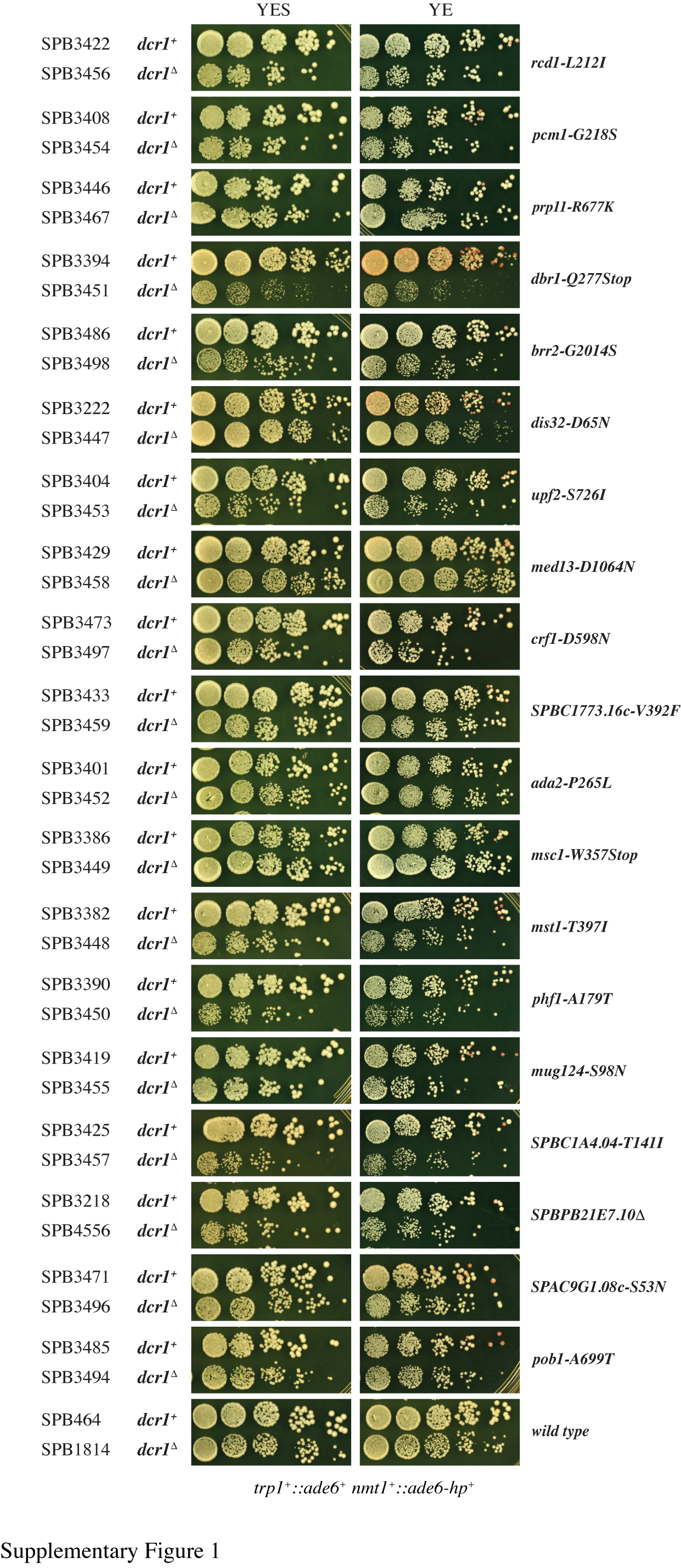

Supplement: S1 Fig — Serial dilution assays showing the ade6+ silencing phenotypes (red color when grown on YE plates) in the respective mutant cells. Silencing is not observed in the absence of Dicer (dcr1Δ). Cells are mst2+ but harbor mutations in individual genes as indicated. (TIF) [file pgen.1009645.s001.tif]

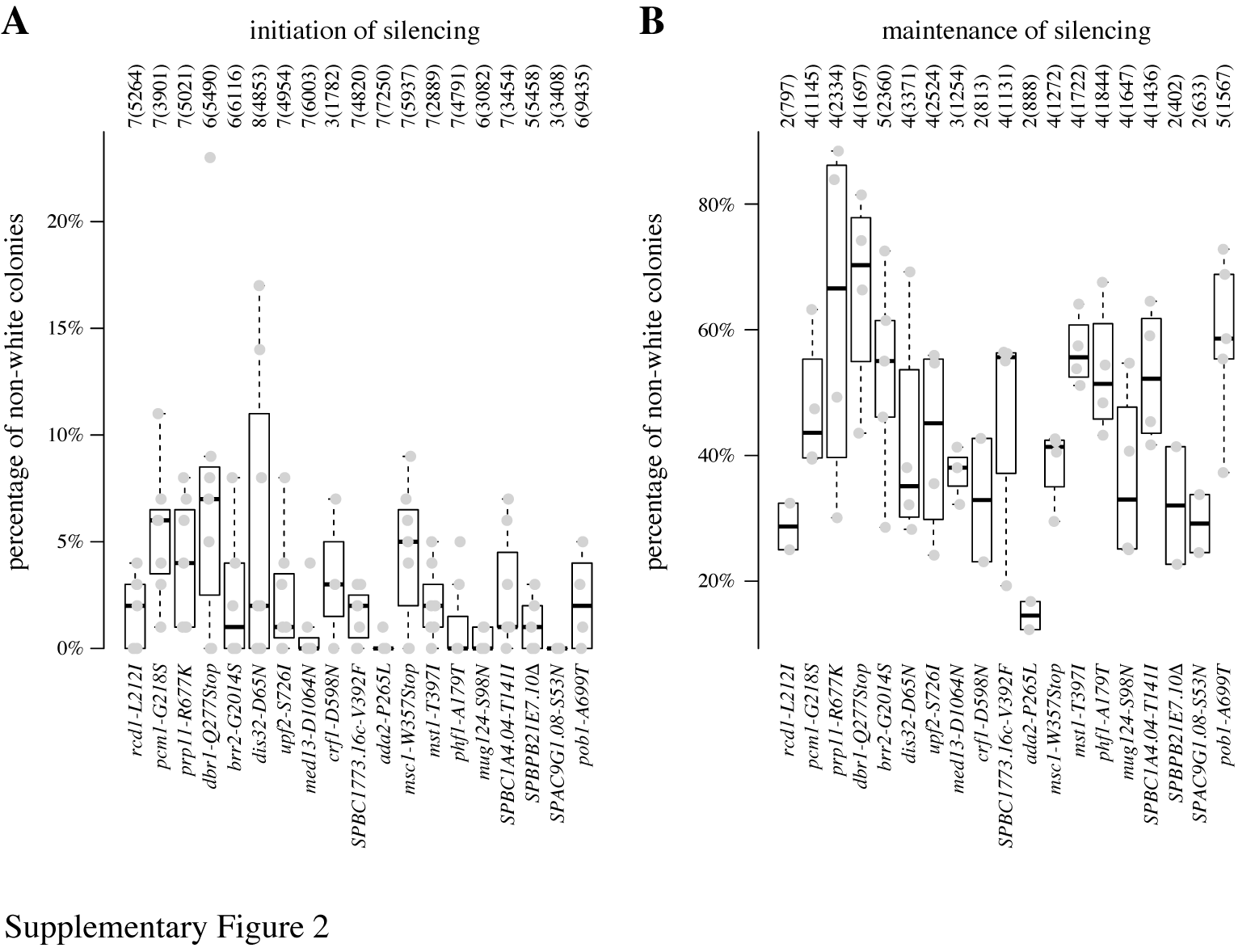

Supplement: S2 Fig — Comparison of initiation (A) and maintenance (B) of ade6+ silencing in the mutant cells as indicated (trp1+::ade6+, nmt1+::ade6-hp+). Multiple individual originator colonies (white or red in A or B, respectively) were spread to single cell density on YE plates to assess initiation/maintenance of the silencing phenotype. Number of originator colonies and total counted number of colonies (in brackets) are indicated on top of the graphs. (TIF) [file pgen.1009645.s002.tif]

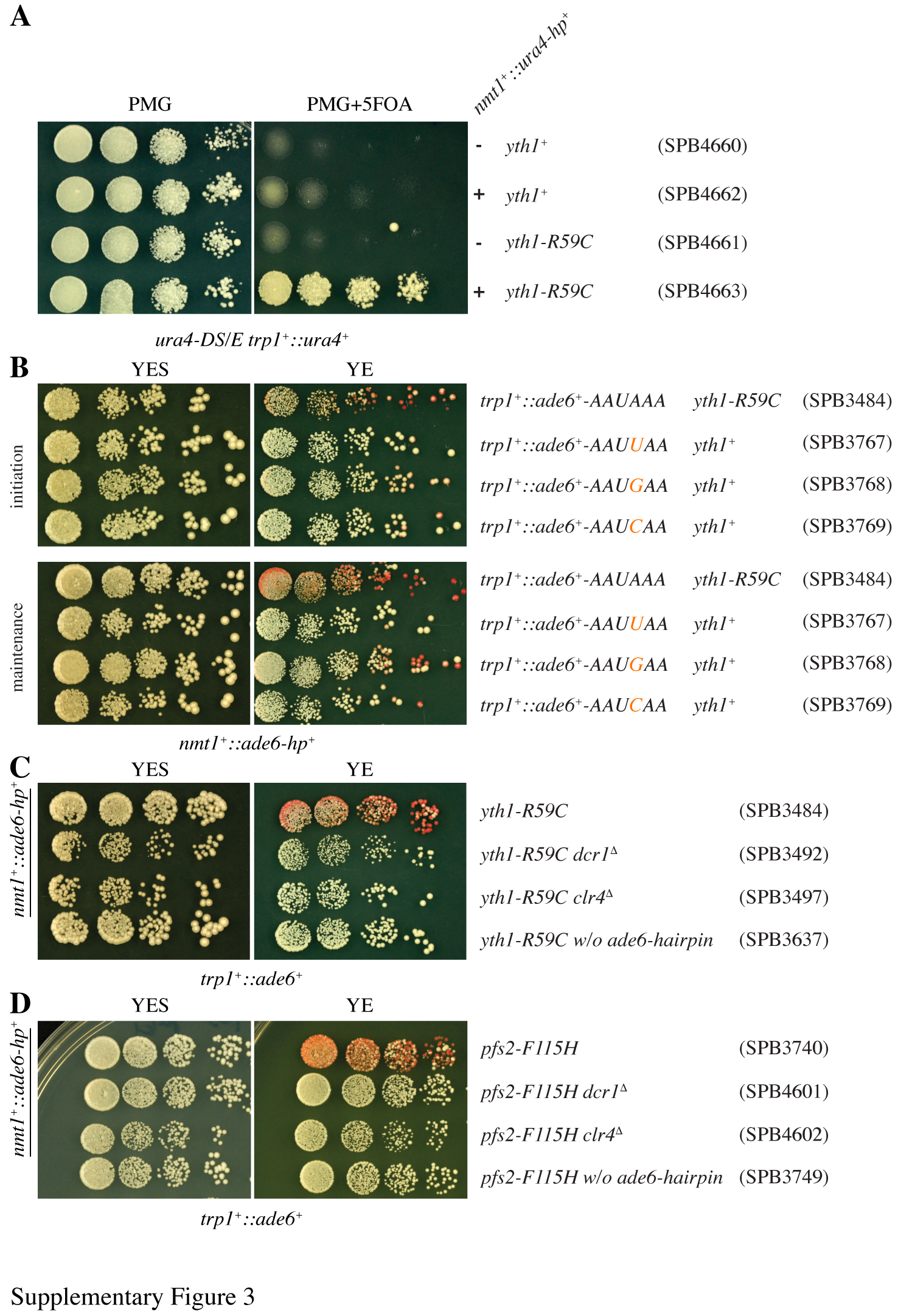

Supplement: S3 Fig — (A) RNAi-directed silencing in yth1-R59C cells is not unique to the trp1+::ade6+ silencing reporter. The yth1-R59C mutation was introduced into cells that express synthetic ura4-hp siRNAs instead of ade6-hp siRNAs, and a trp1+::ura4+ instead of a trp1+::ade6+ reporter. ura4DS/E denotes a partial deletion of the endogenous ura4+ gene. Silencing of the ura4+ reporter was assessed by growth in the presence or absence of 5-FOA (which is toxic to ura4+ expressing cells). Note that 5-FOA resistant colonies did only form in the presence of ura4-hp siRNAs and simultaneous mutation of yth1+. (B) Silencing assays showing the degree of ade6+ silencing in cells harboring mutations in yth1+ (yth1-R59C) or at the 4th position in the PAS of the ade6+ reporter gene. See Fig 3E for a quantification of the initiation of silencing rates in the PAS mutants. (C) Silencing assay demonstrating that trp1+::ade6+ silencing in yth1-R59C cells depends on RNAi (dcr1Δ, w/o ade6-hairpin) and H3K9 methylation (clr4Δ). (D) Silencing assay demonstrating that trp1+::ade6+ silencing in pfs2-F115H cells depends on RNAi (dcr1Δ, w/o ade6-hairpin) and H3K9 methylation (clr4Δ). (TIF) [file pgen.1009645.s003.tif]

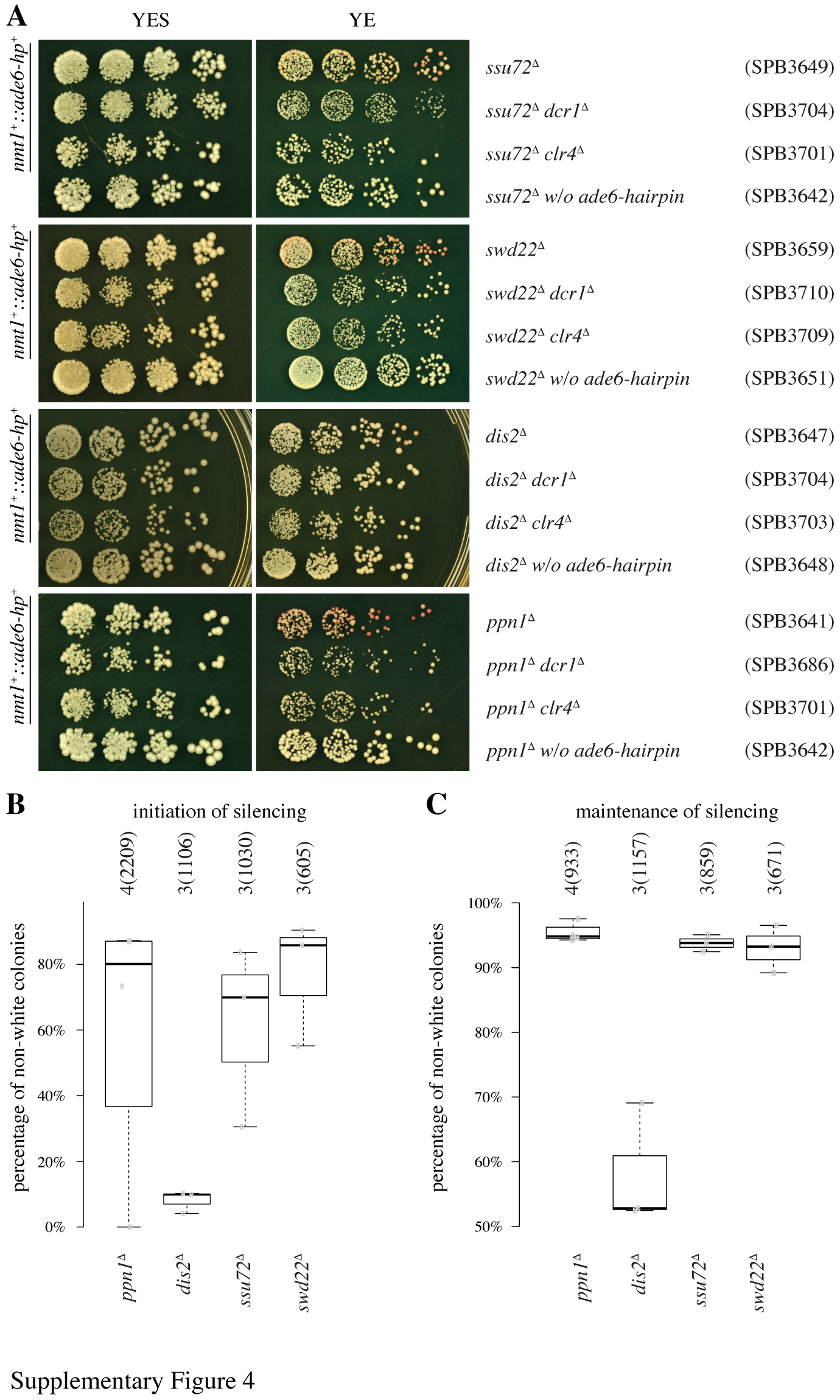

Supplement: S4 Fig — (A) Silencing assays demonstrating that trp1+::ade6+ silencing in CPF phosphatase module mutant cells (ssu72Δ, swd22Δ, dis2Δ, ppn1Δ) depends on RNAi (dcr1Δ, w/o ade6-hairpin) and H3K9 methylation (clr4Δ). (B and C) Comparison of initiation (B) and maintenance (C) of ade6+ silencing in CPF phosphatase module mutant cells (trp1+::ade6+, nmt1+::ade6-hp+). Multiple individual originator colonies (white or red in B or C, respectively) were spread to single cell density on YE plates to assess initiation/maintenance of the silencing phenotype. Number of originator colonies and total counted number of colonies (in brackets) are indicated on top of the graphs. (TIF) [file pgen.1009645.s004.tif]

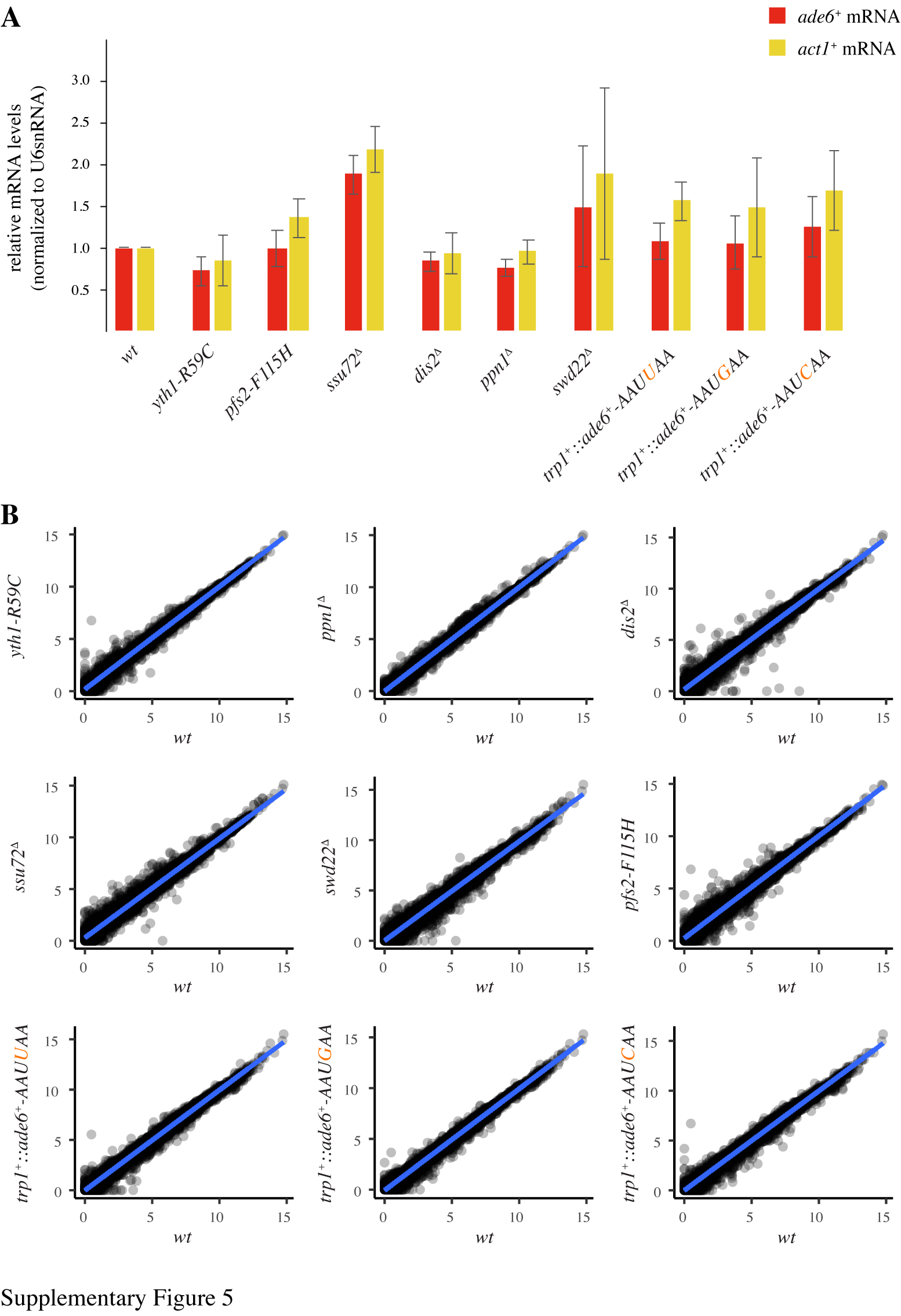

Supplement: S5 Fig — (A) Quantitative RT-PCR with primer pairs amplifying ade6+ or act1+ mRNAs in the respective mutant strains, which do not express any ade6+ siRNA. mRNA levels were normalized to U6snRNA and are shown relative to the levels measured in wild-type cells. Error bars indicate standard deviation, n = 3 independent biological replicates. (B) Pairwise comparisons of gene expression (RNA-seq) between wild-type and CPF or trp1+::ade6+ PAS mutant strains. Cells did not express primary ade6-hairpin siRNAs. Fragments per kilobase million (FPKMs) were calculated and averaged over three replicates. Each scatterplot depicts log2-transformed FPKM values of the wild-type against one of the mutants. (TIF) [file pgen.1009645.s005.tif]
