# Supplementary material for: An enhancer screen identifies new suppressors of small-RNA-mediated epigenetic gene silencing
Source: PLoS Genet. 2021 Jun 22;17(6):e1009645. doi: 10.1371/journal.pgen.1009645 (PMC8253403; doi:10.1371/journal.pgen.1009645)
Supplement: Attachment — Submitted filename: Point-by-point Response_Shimada_Final.docx [file pgen.1009645.s019.docx]

**Manuscript PGENETICS-D-20-01839, point-by-point response to reviewers’ comments**

We are delighted about the positive and constructive feedback that we have received from the reviewers that have evaluated our manuscript. We are confident that we have addressed all the points raised to their satisfaction.

Because of the additional data, our revised manuscript contains one more main figure and 5 supplementary figures.

Below we will respond to the reviewers’ comments point-by-point (our responses are in blue).

*Reviewer #1: It is well known that RNAi machinery is critical for heterochromatin formation at fission yeast centromeres as siRNAs recruit CLRC (Clr4 methyltransferase complex) in cis. However, it has been reported that siRNAs cannot act in trans to trigger heterochromatin at targeted euchromatic locations with the exception of genetic backgrounds that allow such process (e.g. paf1, mst2, mlo3). In this ms. Shimada and colleagues perform a forward genetic screen that identify suppressors of the “trans-acting” small-RNA-mediated epigenetic silencing. The system, already used by the authors in previous papers, consists of a reporter strain that expresses an RNA hairpin on chromosome I that is complementary to an ade6+ reporter gene on chromosome II. In a wild type “naïve” condition, colonies are white (i.e. ade6+ ON). However, if the tester strain acquires enabling mutations yeast colonies become red (i.e. ade6+ OFF), which allows the authors to find 20 novel mutants for genes involved in RNA processing, regulation of transcription, etc. Of outstanding interest, a mutant in Yth1, a component of the conserved CPF (Cleavage and Polyadenylation Factor), display the highest levels of silencing. Such result is counterintuitive since CPF has been recently found to be involved in heterochromatin formation by an RNAi-independent mechanism. The authors also report that mutations in non-essential genes of CPF complex allow trans-silencing, proposing a model in which defective RNA processing delays nascent transcript release from chromatin, allowing sufficient time for the siRNA-guided RITS to recruit CLRC. In summary, this study is in principle interesting for the fission yeast and heterochromatin communities. However, I think that more experimental evidence is required to support the proposed model. In addition, essential controls are missing in some figures. Please find my comments and suggestions below.*

*Major issues:

_Figure 1D: Some critical controls are missing such as wild-type (with or without nmt1:ade6-hp+) and the identified mutants, in particular yth1-R59C, lacking the hairpin. This data must be included to rule out any indirect effect (e.g. mutants displaying red color per se). This information can be presented as dilution assays given the number of strains involved.*

We are assuming the reviewer refers to Figure 1C. The design of our screen included various steps to control for indirect effects (see text and Figure 1B) and we are happy to show actual data in the revised version of our paper as requested:

- We have added wild type colonies (cells expressing the *ade6*+ hairpin/siRNAs), showing the white phenotype, to Figure 1A. Dilution assays of wild type cells are also part of the new Supplementary Figure 1.

- The requested data for *yth1-R59C* was already provided in our initial submission (Figure 2B).

- For the other 19 mutants we provide dilution assays in the presence or absence of siRNAs in Supplementary Figure 1.

- We also provide initiation and maintenance rates for all the mutants in Supplementary Figure 2.

*_Figure 2E: Can the authors rule out a mere redistribution of heterochromatin from other locations to the trp1::ade6+ region? For example, in Shelterin mutants, heterochromatin released from telomeres can relocate to centromeres thus suppressing RNAi mutants (Tadeo et al., 2013 -Jia Group)*

Yes, we can. Figure 2B shows that *trp1::ade6^+^* silencing is strictly dependent on the presence of the ade6-hairpin (siRNAs). Furthermore, silencing is accompanied by the production of secondary siRNAs (see new data in Figure 2F and Figure 4C). Thus, a mere redistribution of heterochromatin in *yth1* mutants as the underlying cause of the observed silencing can be ruled out.

*_Figure 3E: The authors shall also show colony color pictures directly comparing yth1-R59C to PAS A4 mutants. This is a critical for ruling indirect effects caused by CPF mutants. I would expect similar levels of silencing between these mutants if the authors’ model is correct. Also, the levels of H3K9me2 in PAS A4 mutants as compared to yth1-R59C needs to be shown.*

We do not agree with the reviewer’s assumption. Levels of silencing must not necessarily be the same if comparing point mutations in CPF with nucleotide substitutions in the PAS, because none of these are loss of function mutations. This is evident from Figures 5C and 2C/3E, which show that the impact on pre-mRNA cleavage and initiation of silencing is greater in the Yth1 mutant compared to PAS mutants, respectively. To complement this, we show the requested dilution assays of *yth1-R59C* and PAS mutant cells side-by-side in Supplementary Figure 3B of the revised manuscript. The data we provide in the paper rule out indirect effects (see responses to other comments).

*_In addition, ChIP analyses for Yth1 and Yth1-R59C is needed to rule out any indirect effect. I would expect lower enrichment for the mutant at the trp1:ade6+.*

We do not expect CPF recruitment to the transcribed locus to be affected. Even if it did, it would not rule out indirect effects. As already stated above, it is RNAi-dependence of *trp1::ade6^+^* repression that rules out indirect effects (siRNAs are providing the specificity).

*_Citations are missing as follows:

Lines 241-243: Please cite Lee et al. 2020 (PMID: 32101745), since this paper also identified CPF factors involved in heterochromatin formation.*

Thank you! We cite this paper in the revised manuscript.

*Introduction: The section describing siRNA becoming potent mediators of RNAi-mediated epigenetic silencing in mutants such as mlo3∆ is missing an important reference. The authors must acknowledge a paper by Reyes-Turcu et al. NSMB 2011 (PMID: 21892171; Grewal group) that was first to show that cells lacking mlo3 show hairpin-induced heterochromatin assembly (see fig S7). Reyes-Turcu et al also showed that treatment of cells 6-AU enhances hairpin-induced heterochromatin assembly. The system is very similar to that used later in Kowalik et al., 2015.
The authors also discuss an RNAi positive feedback loop. This positive feedback loop was first discovered by Noma et al (PMID: 15475954) and then further described by Sugiyama et al (PMID: 15615848). These papers should be referenced along with work by the authors.*

We apologize for not mentioning this work. These papers are cited in the revised manuscript.

*Minor comments:

_Lines 188-190: How mst2+ strains were obtained? While some information is available in Materials and Methods, it is important to point out that strains are not coming from crosses, so it is not mere maintenance of silencing state carried from the mst2∆ background.*

The strains were generated by transformation. This information is provided in the Materials & Methods section.

*_Fig. 2C. Are the other mutants affecting initiation of silencing as well? Any additional data would be useful in a supp. figure. Similarly, initiation of silencing rates in CPF mutants would be helpful as well.*

The *yth1^+^* mutant clearly shows superior initiation rates. We provide initiation and maintenance rates for all the mutants in Supplementary Figure 2 of the revised manuscript. Initiation and maintenance rates of CPF mutants are shown in Supplementary Figure 4B and C.

*_Fig. 2E. p values should be written as “0.01” instead of “0,01” and so on.*

Corrected. *_Fig. 3A: I wonder how CPF mutants were obtained. Where these strains obtained from crosses involving an already ade6+ silenced? Please clarify.*

We assume this reviewer is talking about Figure 4A (Figure 4B in the revised MS). No, these strains were obtained by transforming a KanR resistance cassette deleting the respective genes. Thus, *ade6^+^* had not been previously silenced.

*_Fig. 4B-D. If data is available for pfs2 mutant, that should be included.*

Pfs2 mutant stains were not included in these analyses.

*_Fig. 4C. Is the reverse oligo annealing after the cleavage site? Please clarify.*

Yes. As indicated in the scheme, the rev primer is annealing after the cleavage site. We have added a scissors symbol in what is now Figure 5A to be clear.

*_Table S3 and S4: please indicate in which figure/s the strains or oligos have been employed. That will help the reader.*

We have updated Supplementary Table S4 with that information. For Supplementary Table S3 it is obvious.

*Reviewer #2: The role of small interfering non-coding RNAs in promoting de novo heterochromatin formation in cis was described almost two decades ago using the fission yeast Schizosaccharomyces pombe. Since then, many groups have dissected in detail many of the different components that are required for establishing RNAi-dependent heterochromatin domains at non-coding repeats.

Interestingly, contrary to the effect observed in cis, it has consistently been reported that in wild-type cells, siRNAs derived from a hairpin construct (or alternatively from a source gene inserted within heterochromatin repeats) do not lead to effective transcriptional silencing of a complementary target sequence in trans. However, this refractory effect was shown to be suppressed in the presence of specific enabling mutations, such as those in the Paf1 complex or the mRNA export factor Mlo3.

In this manuscript, Shimada et al. report the identification of 20 novel mutant alleles that enable hairpin-derived siRNAs to mediate transcriptional silencing of a target ade6 gene in trans. The authors followed the same strategy that led to the identification of Paf1C mutants in a previous study (Kowalik et al., 2015) but here, to maximise the chances of obtaining trans silencing-enabling mutations, the authors performed their EMS screen in a mst2D background.

Of 20 identified alleles, the authors decided to focus on yth1-R59C, encoding a mutant of the CPF complex subunit Yth1, which showed the strongest silencing phenotype. Indeed, yth1-R59C cells exhibited a remarkably high rate of silencing establishment. Furthermore, using a mutagenized plasmid library-based strategy, the authors identified additional mutations in yth1 that enable siRNA-mediated silencing. Modelling the identified yth1 mutations on the structure of human CPF revealed that R59C has the potential to disturb the interaction with adenosine at position 4 of the pre-mRNA PAS. Consistent with this hypothesis, the authors showed that strains harbouring mutations in A4 of the ade6 PAS are able to establish siRNA-mediated silencing in the absence of yth1-R59C or other enabling mutations.
A mutation in Pfs2 (another CPF component) was also found to allow siRNA-mediated silencing to occur in trans, and a similar effect was proposed to be achieved upon deletion of several CPF phosphatase module components.

Lastly, in an attempt to consolidate their previously-proposed kinetic nascent transcript processing model for the inhibition of siRNA-mediated de novo heterochromatin establishment, the authors show that in strains harbouring yth1-R59C or A4 mutations, ade6 pre-mRNA cleavage is reduced.

The manuscript is in general well-written and easy to follow. Even though mutations that enable siRNA-mediated silencing in trans have been described before, this work is of interest as it certainly provides a list of novel enabling mutations, some of which occurred in uncharacterized factors or components with biological roles not previously associated with siRNA-mediated silencing. However, further mechanistic insights should be provided regarding the role of yth1 mutations in enabling siRNA-mediated silencing through the previously proposed kinetic nascent transcript processing model, as the presented experiments only further solidify the correlation between siRNA-mediated silencing establishment and levels of non-cleaved pre-mRNA.

Major points:

1. In the abstract, the authors state: ‘Our results provide direct evidence for a kinetic nascent transcript processing model‘, yet experiments presented in Figure 4 only show that yth1-R59C cells exhibit reduced cleavage of ade6 pre-mRNA. No evidence of unprocessed transcripts being retained on chromatin is provided. The authors should directly test if an increase in chromatin-bound unprocessed ade6 transcripts in yth1-R59C cells is associated with the observed high silencing establishment rate. Can the high silencing establishment frequency of yth1-R59C be suppressed by engineering pre-mRNA cleavage (e.g. ribozyme insertion as used in S. cerevisiae Mol Cell 2003 12:711 ) and release from chromatin?*

We are grateful for this comment and agree with the reviewer that we overstated our findings with regard to the kinetic nascent transcript model. While our results further support such model, we don’t directly demonstrate this in the paper indeed. We have previously used the suggested ribozyme strategy in the context of an enabling mutation in the *paf1*^+^ gene (Kowalik et al, 2015). To address this critique, we have generated new strains with the wild type and mutant ribozyme sequences inserted into *ade6*^+^ in the *yth1-R59C* background. Below we show silencing assays that we have performed with two independent active and inactive ribozyme insertion strains (ade6Rz and ade6RzM, respectively).

Unfortunately, the results that we have obtained are not conclusive. Although silencing might be partly suppressed by the ribozyme insertion, wild type and mutant ribozyme strains look similar, which does not allow us to conclude that engineering of pre-mRNA cleavage with ribozymes in *yth1-R59C* cells would suppress silencing establishment.

Because we provide indirect evidence only for the nascent transcript processing model, we have changed our statements in the text accordingly.

*2. Although the establishment of siRNA-mediated silencing at a complementary sequence in trans has previously been shown to correlate with secondary siRNA generation at the target site, no experiments aimed at detecting those were performed here. Confirming the presence of secondary siRNAs is of special importance in yth1-R59C cells, since the authors performed substantial follow-up experiments to elucidate the mechanism by which this mutation enables silencing.*

This is an excellent point. We have generated small RNA libraries of the CPF subunits studied in our paper and subjected them to next-generation sequencing. As expected, secondary *ade6+* siRNAs are generated in *yth1-R59C* cells. This data is shown in Figure 2F of the revised manuscript. Secondary *ade6+* siRNAs are also generated in Pfs2 and phosphatase module mutants. This data is shown in Figure 4C of the revised manuscript.

*3. Line 303-305: ‘CPF mutants that we have investigated in this study phenocopy wild type cells, i.e. neither growth nor gene expression is noticeably affected.’

- There seems to be a small but noticeable difference in colony size when comparing yth1 mutants to wild-type cells. These differences can be observed in YES plates of Figure 3B and Figure 4A (also for dis2D and ssu72D mutants).*

We agree that mutant colonies are slightly smaller. We have therefore changed this sentence to “A remarkable feature of *CPF mutants that we have investigated in this study is that they by and large phenocopy wild type cells, i.e. neither growth nor gene expression is largely affected. “*

To further corroborate this notion, we have included RNA-seq and RT-PCR data in Supplementary 5 of the revised manuscript.

*- Quantitative analyses of ade6 mRNA levels (without ade6-hp) in yth1-R59C, A4 PAS, and phosphatase mutants compared to wild-type cells are not shown in this manuscript. Although a strong change in ade6 mRNA levels is not expected in yth1-R59C and A4 mutants by looking at the phenotypes shown in Figure 2B (no ade6-hp, dcr1+) and 3E, ade6 mRNA levels should be quantitatively assessed in these conditions and compared to levels observed in wild-type cells. Figure 4B shows differences in cleavage among mutants but does not compare total levels. Figure 4C (bottom) suggests that ade6 mRNA levels in mutants are equal to wild-type but a loading control was not included in this experiment.*

We included Supplementary Figure 5 in the revised version of our manuscript, which shows RT-qPCR measurements of *ade6*+ and *act1*+ mRNA levels in the respective mutant cells, as well as global transcriptome measurements by RNA-seq. The data supports our notion that steady-state mRNA levels are not greatly affected in the CPF mutants.

*- For phosphatase mutants, in addition to quantitatively assessing ade6 mRNA levels, a phenotype (colour) assay should be shown in the absence of ade6-hp or alternatively in the presence of ade6-hp but in a dcr1D background. Data shown in Figure 4 are not enough to support the conclusion that these mutations enable siRNA-mediated silencing (this is of importance given that these mutations did not come up in the screen which excluded mutants in the adenine pathway).*

We provide the color assays for the phosphatase mutants in *dcr1Δ and clr4Δ* backgrounds, as well as in the absence of the ade6-hairpin in Supplementary Figure 4A of the revised manuscript.

*Other points:

- Figure 4 and text lines 331/337 refer to yth1-G59C instead of yth1-R59G which made the respective figure and text highly confusing.*

Thanks. We have corrected this in the revised manuscript.

*- Some pink colonies are formed in p-yth1+-containing cells in YE medium. Is this due to differences in plasmid stability among clones?*

Yes, indeed, in rare cases pink colonies appear when *yth1^+^* is expressed from a plasmid. Plasmid stability is a possible explanation.

*- Given that phosphatase mutants do not affect pre-mRNA cleavage, could the authors speculate on how these mutations lead to siRNA-mediated silencing?*

We hypothesize that the inability to dephosphorylate their (unknown) substrates enables the silencing. Modulation of epigenetic silencing through phosphorylation is an intriguing possibility that we mention in the discussion of our manuscript.

*- The use of the term ‘tester strain’ is at times confusing as it is unclear whether this refers to the original mst2+ tester strain used in Kowalik et al. (2015) or the mst2D strain used in this study. I suggest adding mst2+ or mst2D in between brackets every time ‘tester strain’ is mentioned.*

Thanks, we have amended the text accordingly.

*- Figure 2C/D/E. Please indicate genotype either in panels or in legend (no mention of ade6-hairpin is made for those panels).*

Thanks. We have added the precise genotypes to the figure legend.

*- Including the silencing establishment frequency for each of the identified mutant alleles (both in mst2D and mst2+ backgrounds) in Table 2 would be helpful to the community.*

That is a good idea. In response to reviewer 1, we generated Supplementary Figure 2, which shows the initiation and maintenance rates for all mutants in the *mst2^+^* background. We refrain from providing these numbers for the *mst2Δ* background because these strains underwent mutagenesis, hence secondary mutations could influence these numbers.

*- Line 291 - Forgoing > Foregoing?*

Corrected.

*- Figure 4C/D: Diagrams illustrating primer locations (C (top) and D) are confusing at they seem to indicate that the amplicon in D is longer than C (top).*

The scheme is not drawn to scale and the forward primers used are not the same. We have changed the color of the primer symbols in the figure. Furthermore, primers are further specified in Supplementary Table S4.

*- Although a reference is provided, given the importance of the procedure for this study, it would be appropriate to fully describe the computational pipeline followed to identify SNPs, especially as it is indicated that an ‘adapted’ pipeline was used.*

We have added the full description to Material & Methods of the revised manuscript.

*Reviewer #3: Heterochromatin is essential for genome stability and gene expression. Small interference RNAs (siRNAs) have been shown to plays an important role in peri-centromeric heterochromatin, which contains repetitive DNA, in fission yeast. However, the protein-coding genes in euchromatin are resistant to siRNA-mediated heterochromatin formation. The mechanism used for counteracting heterochromatin assembly remains largely unclear. Through an elegant genetic screen, Shimada et al. identified more than twenty mutant alleles that enable de novo formation of heterochromatin at a protein-coding gene in euchromatin. By further characterizing a novel factor they identified, the pre-mRNA cleavage factor Yth1, they provides new insight into how small-RNA-directed de novo formation of heterochromatin in euchromatin is inhibited. This work is scientifically interesting and significant. The experiments were in general well designed and carefully executed, and most of the results are convincing. There are several concerns as listed below.

Major:

1. There is a concern about the indirect effect on the expression of the ade6 reporter used in the genetic screen in the mutant backgrounds. The study will be strengthened by confirming the de novo heterochromatin formation using growth assays having a different reporter.*

Replicating the entire study with another reporter gene is neither feasible nor would we consider it necessary because our screen was designed such that indirect effects that can cause a red color phenotype are filtered out (Figure 1B and text). Because silencing in our final hit list is strictly dependent on *ade6*+ siRNAs, indirect effects can be confidently ruled out. In addition to the data already provided in the original submission, we have included additional controls in Supplementary Figures 1, 3, 4, and 5.

To nonetheless show that silencing is not gene specific, we have generated new *yth1-R59C* strains using the *ura4^+^* reporter/ura4-hairpin system. 5FOA silencing assays are shown in Supplementary Figure 3A of the revised manuscript.

*2. Figure 1C should have a wild type control.*

We show wild type colonies as part of panel A, in comparison to the mutants displayed in panel C, in the revised version of the paper.  *3. The study would be benefited by analyzing mutants with de novo heterochromatin formation in the clr4 mutant background using same growth assays. This would further confirm that the de novo heterochromatin formation depends on H3K9 methylation.*

We have deleted *clr4^+^* in the *yth1-R59C, pfs2-F115H* and phosphatase mutant strains. Silencing is lost in these mutant backgrounds, further confirming heterochromatin-mediated repression. We show these results in Supplementary Figures 3 and 4 of the revised paper.

*4. It would be interesting to compare the de novo heterochromatin silencing in these mutants with the native pericentromere silencing, such as H3K9 methyation and RNA transcription level of the reporter in the two regions.*

Silencing within pericentromeric, constitutive heterochromatin is evidently very robust (essentially no variegation). In contrast, there is a wide range of silencing (variegation) across the newly identified mutants that enable de novo silencing, which we believe is documented adequately in our manuscript.

*5. In Introduction, Line 94, the statement “Whereas siRNAs originating from heterochromatic repeats function well in cis to sustain H3K9 methylation, they do not act in trans to mediate de novo formation of heterochromatin at complementary sequences in wild type cells “ is inaccurate. It has been shown that siRNAs can act in trans to induce heterochromatin formation at complementary sequences in pericentromeres in Chromosome III in wild type fission yeast cells (Haijin He, et al. Cell Reports, 2016).*

Thanks. We meant to say “Whereas siRNAs originating from heterochromatic repeats function well in cis to sustain H3K9 methylation, they do not act in trans to mediate de novo formation of heterochromatin at complementary protein-coding genes outside centromeric repeats in wild type cells“. We have amended the text accordingly.

*Minor

1. The media used in this paper should be listed in the Materials and Methods section.*

As indicated in the Figures, standard YES or YE media was used.

*2.Line 570, it should be “wild-type”, not “wildtype”.*

Thanks, corrected.
